# The role of the bacterial microbiome in the treatment of cancer

**DOI:** 10.1186/s12885-021-08664-0

**Published:** 2021-08-19

**Authors:** Zi-Kun Yu, Rui-Ling Xie, Rui You, You-Ping Liu, Xu-Yin Chen, Ming-Yuan Chen, Pei-Yu Huang

**Affiliations:** 1 Department of Nasopharyngeal Carcinoma, Sun Yat-sen University Cancer Center, 651 Dongfeng East Road, Guangzhou, 510060 People’s Republic of China; 2 Sun Yat-sen University Cancer Center; State Key Laboratory of Oncology in South China; Collaborative Innovation Center for Cancer Medicine, Guangdong Key Laboratory of Nasopharyngeal Carcinoma Diagnosis and Therapy, Guangzhou, 510060 China

**Keywords:** Gut microbiome, Locally resident microbiome, Intratumour microbiota, Cancer treatment, Mechanism, Future research

## Abstract

The human microbiome is defined as the microorganisms that reside in or on the human body, such as bacteria, viruses, fungi, and protozoa, and their genomes. The human microbiome participates in the modulation of human metabolism by influencing several intricate pathways. The association between specific bacteria or viruses and the efficacy of cancer treatments and the occurrence of treatment-related toxicity in cancer patients has been reported. However, the understanding of the interaction between the host microbiome and the cancer treatment response is limited, and the microbiome potentially plays a greater role in the treatment of cancer than reported to date. Here, we provide a thorough review of the potential role of the gut and locally resident bacterial microbiota in modulating responses to different cancer therapeutics to demonstrate the association between the gut or locally resident bacterial microbiota and cancer therapy. Probable mechanisms, such as metabolism, the immune response and the translocation of microbiome constituents, are discussed to promote future research into the association between the microbiome and other types of cancer. We conclude that the interaction between the host immune system and the microbiome may be the basis of the role of the microbiome in cancer therapies. Future research on the association between host immunity and the microbiome may improve the efficacy of several cancer treatments and provide insights into the cause of treatment-related side effects.

## Background

The human microbiome can be considered an organ of the human body and is defined as the microorganisms that reside in the human body, such as bacteria, viruses, fungi, and protozoa, and their genomes [1]. Recently, the relationship between the human microbiome, especially the gut microbiome, and human disease has attracted increasing attention. Probiotics, defined as “live microorganisms that, when administered in adequate amounts, confer a health benefit on the host [2], have been shown to participate in the modulation of human metabolism by influencing several intricate metabolic pathways [3]. The gut microbiome is an indispensable part of the human microbiome and refers to the enormous number of microorganisms resident in the gastrointestinal tract. Dysbiosis of the gut microbiome, in which there are abnormalities in the types and number of organisms present in the natural microflora of the host, has been shown to be associated with digestive, neurologic, metabolic, respiratory and several other illnesses [4, 5]. Cancer is a leading cause of death worldwide [6]. The development of some cancers has been found to be associated with specific bacterial or viral infections [7–12]. Moreover, researchers have found that the treatment-related toxicity of cancer therapy can be mediated by different constituents of the human microbiome [13–16]. A series of studies have demonstrated that the gut microbiota can influence the host immune response to tumours and strongly affect the response to cancer treatment, especially immune checkpoint blockade, in both clinical cohorts and mouse models [17–26]. Additionally, the association between locally resident microbiota or intratumour microbiota and carcinogenesis and the effect of cancer therapy is a current focus of microbiome research. The locally resident microbiota is likely to be another useful prognostic factor for cancer patients [11, 27–29]. However, understanding of the association between the host microbiome and the cancer treatment response is limited to specific cancers, suggesting the necessity of research into this association in other cancer types. Several cancers and treatments have been found to be influenced by the microbiome, especially the bacterial microbiome, by next-generation sequencing, which helps identify the species and quantity of these microorganisms. A timely update and summary of the developments in research into the microbiome and cancer treatment are necessary to promote future studies. Although some reviews have covered the role of the human microbiome and cancer treatment [25, 30], there is still no clear direction for future research. With the development of knowledge of the microbiome, more detailed mechanisms and methods to modulate the microbiome have been revealed, which need to be summarized and applied in future research. Herein, we attempted to determine future direction of research in this field and develop a plan for future research by studying the functions and mechanisms of the host microbiome. A comprehensive literature search was performed to identify, collate and analyze previously published research. Studies published before November 2020 were included if they were identified by the search strategy and met the selection criteria. The literature search was conducted using the PubMed, Web of Science, Embase and Cochrane databases, and search terms used were “cancer”, “treatment response”, and “microbiome”. Only studies on bacterial microbiota were included and discussed in our review. The reference lists of the included papers and review articles were also searched. We discuss the most recent reports, in particular a breakthrough report on the association between the microbiota and several cancer therapies, to explore the potential role of the gut microbiota and locally resident microbiota in cancer therapy. The reported mechanisms of this relationship, such as metabolism, the immune response and the translocation of the microbiome, is also discussed to promote future research into this association in other cancer types.

## Microbiome and cancer therapy

Research into the molecular mechanisms of carcinogenesis and the progression and metastasis of cancer has yielded several cancer therapies, such as surgery, radiation therapy, chemotherapy, targeted therapy, immune checkpoint blockade and hormone therapy. Researchers believe that cancer can arise due to the attenuation of immunosurveillance and the development of immunological tolerance to tumour-derived antigens [31]. Several studies have shown that the gut microbiota can modulate the host immune response to tumours [17]. The potential role of the gut microbiota as a biomarker for predicting the efficacy of cancer treatment has attracted the interest of clinical researchers. For locally resident microbiota, many studies on the complex interaction between the gut and locally resident microbiota have recently shown that locally resident microbiota can be a prognostic factor of cancer treatment [11, 27–29]. Herein, we separately discuss the impact of the locally resident microbiota and the gut microbiome on cancer and cancer therapy.

## Cancer therapy and the gut microbiome

### Immune checkpoint inhibitors (ICIs)

The utilization of immune checkpoint inhibitors (ICIs) in cancer treatment, including monoclonal antibodies targeting the programmed death receptor (PD-1), ligand of programmed death receptor (PD-L1) and cytotoxic T lymphocyte-associated protein 4 (CTLA-4) receptor, is considered a revolution in cancer therapy that changes the poor prognosis of many malignancies and is widely used in the treatment of advanced-stage cancer [32–39]. However, primary and secondary resistance to cancer treatment can seriously influence patient outcomes and remains a challenge [40]. Recently, the gut microbiota was found to have a strong impact on tumour response to ICIs in both clinical cohorts and preclinical mouse models [18–26]. Some of the earliest work on the influence of gut microbes on the efficacy of ICIs for several cancers was performed in preclinical mouse models [22, 41]. Researchers have demonstrated that tumours of the same mouse strain purchased from different suppliers with different gut microbiomes have distinct responses to ICIs that target PD-1 for melanoma [22]. Another study was conducted to test whether the relationship between the response to CTLA-4 monoclonal antibodies and the gut microbiome was the same as that in anti-PD1 therapy and it was found that *Bacteroidales* play a key role in the effects of tumour immunity induced by the blockade of CTLA-4 [41]. In these studies, mice with a “favourable” gut microbiome had a better response, which may result from the enhancement of the T cell response via the activation of antigen presenting cells (APCs), such as dendritic cells.

Recent clinical studies also suggested that dysbiosis of the gut microbiome can induce resistance to ICIs, highlighting the distinct role of the gut microbiome in regulating ICI efficacy and side effects [13, 19–21]. Subsequent research on the relationship between immune checkpoint blockade and the gut microbiota concentrated on faecal microbial transplantation (FMT) in murine models to verify the results from human studies [18, 20]. Studies on the association between different gut microbiomes and the efficacy of cancer immunotherapy are summarized in Table 1. *Bacteroidetes* was found to be a biomarker of nonresponders to immune checkpoint inhibitors in metastatic melanoma (MM) patients in several studies [13, 14, 20, 41] and may decrease the response rate and attenuate systemic and antitumour immunity, leading to a decreased risk for local inflammation, such as ICI-induced colitis. Nevertheless, some *Bacteroidetes*, such as *Bacteroides thetaiotamicron* and *B. caccae,* are associated with an effective therapeutic response [21, 22, 41]. *Faecalibacterium*, *Bifidobacterium* and *Ruminococcaceae* can improve the therapeutic response to ICIs, while *Faecalibacterium* and other *Firmicutes* may lead to a higher risk for ICI-induced colitis [18, 19, 21, 24]. In non-small-cell lung cancer (NSCLC) and renal cell carcinoma (RCC), *Akkermansia muciniphils* and *Alistipes* are markers ICI responders [18]. Pretreatment antibiotics (ATBs) have been found to have a negative impact on ICI efficiency due their impact on the diversity of the gut microbiome and lead to secondary dysbiosis [18, 43, 44]. For instance, Routy [18] found that progression-free survival (PFS) and overall survival (OS) times were significantly shorter in a group of patients treated with ATBs for NSCLC, RCC and urothelial carcinoma (UC) and anti-PD1-based immunotherapy. The median PFS and OS times for the ATB group were 3.5 months and 11.5 months, respectively, while the median PFS and OS times for patients who did not receive ATBs were 4.1 months and 20.6 months, respectively (*p* = 0.017 for PFS and *p* < 0.001 for OS). These studies suggest a strong correlation between the intestinal flora and the efficacy of tumour immunotherapy.

**Table 1:** Clinical and preclinical studies about the association between gut microbiome and the host response to immune checkpoint inhibitors sorted by the category of bacteria

Gut microbiome categories	Mice model, or patients	Impacts	References
*Bacteroidetes* *Bacteroides* *B. fragilis* *B. thetaiotaomicron* *B. caccae* *Bacteroides thetaiotamicron* *Bacteroidales*	Antibiotic-treated or germ-free mice with MCA205 sarcomas receive Anti-CTLA-4(Ipilimumab) therapy	Improved response and reduced colitis	Vetizou et al. (2015) [41]
	MM patients receive Anti-CTLA-4(Ipilimumab) therapy	Reduced the risk of ICIs induced colitis	Dubin et al. (2016) [14]
	Adult MM patients receive Ipilimumab, Nivolumab Ipilimumab plus nivolumab, pembrolizumab (Anti-CTLA-4 or Anti–PD-1 or combination of Anti-CTLA-4 and Anti–PD-1 therapy)	Predicted effective therapeutic response	Frankel et al. (2017) [21]
	MM patients receive Anti-CTLA-4(Ipilimumab) therapy	Bacteroides decreased therapeutic response and decrease risk of ICIs-induced colitis	Chaput et al. (2017) [13]
	MM patients, germ-free mice with injection of BP melanoma cell receive Anti–PD-1 therapy	Bacteroidales decreased response, and attenuate systemic and antitumor immunity	Gopalakrishnan et al. (2018) [20]
*Bifidobacterium* *B. pseudolongum* *B. longum*	Melanoma mice with distinct commensal microbiota receive Anti–PD-L1 therapy	Delayed melanoma growth, and enhanced CD8 + T cell priming and accumulation in the tumor microenvironment	Sivan et al. (2015) [22]
	MM patients receive Anti–PD-1 therapy	Improved therapeutic response, enhanced tumor control, and improved T cell response.	Matson et al. (2018) [19]
	germ-free or specific-pathogen-free mice with injection of MC38 colorectal cancer cells receive anti CTLA-4 treatment	*B. pseudolongum* (belongs to *B. pseudolongum*) enhanced immunotherapy response through production of the metabolite inosine	Mager et al. (2020) [42]
*Faecalibacterium and other Firmicutes* *Clostridiales Faecalibacterium* *Faecalibacterium prausnitzii*	MM patients , germ-free mice with injection of BP melanoma cell receive Anti–PD-1 therapy	Clostridiales Faecalibacterium and Ruminococcaceae Improved response, enhanced systemic and antitumor immunity	Gopalakrishnan et al. (2018) [20]
	Adult MM patients receive Ipilimumab, Nivolumab Ipilimumab plus nivolumab, pembrolizumab (Anti-CTLA-4 or Anti–PD-1 or combination of Anti-CTLA-4 and Anti–PD-1 therapy)	Predicted effective therapeutic response	Frankel et al. (2017) [21]
	MM patients receive Anti-CTLA-4(Ipilimumab) therapy	Faecalibacterium and other Firmicutes Improved therapeutic response and higher risk of ICIs induced colitis	Chaput et al. (2017) [13]
*Ruminococcaceae*	MM patients, germ-free mice with injection of BP melanoma cell receive Anti–PD-1 therapy	Ruminococcaceae Improved response, and enhanced cancer immunity	Gopalakrishnan et al. (2018) [20]
*Collinsella aerofaciens*	MM patients receive Anti–PD-1 therapy	Improved therapeutic response, enhanced tumor control, and improved T cell response.	Matson et al. (2018)
*Enterococcus faecium*	MM patients receive Anti–PD-1 therapy	Improved therapeutic response, enhanced cancer immunity, and improved T cell response.	Matson et al. (2018)
*Olsenella species*	germ-free or specific-pathogen-free mice with injection of MC38 colorectal cancer cells receive anti CTLA-4 treatment	Improved therapeutic response, enhanced cancer immunity, and improved T cell response.	Mager et al. (2020) [42]
*Lactobacillus johnsonii,*	germ-free or specific-pathogen-free mice with injection of MC38 colorectal cancer cells receive anti CTLA-4 treatment	Improved therapeutic response, enhanced cancer immunity, and improved T cell response.	Mager et al. (2020) [42]
*Holdemania filiformis*	Adult MM patients receive Ipilimumab, Nivolumab Ipilimumab plus nivolumab, pembrolizumab (Anti-CTLA-4 or Anti–PD-1 or combination of Anti-CTLA-4 and Anti–PD-1 therapy)	Predicted effective therapeutic response	Frankel et al. (2017) [21]
*Dorea formicogenerans*	Adult MM patients receive Ipilimumab, Nivolumab Ipilimumab plus nivolumab, pembrolizumab (Anti-CTLA-4 or Anti–PD-1 or combination of Anti-CTLA-4 and Anti–PD-1 therapy)	Dorea formicogenerans enriched only in patients who accepted pembrolizumab and predicted effective therapeutic response	Frankel et al. (2017) [21]
*Akkermansia muciniphil*	NSCLC and RCC patients receive Anti-PD-L1 and Anti–PD-1	Improved therapeutic response	Routy et al. (2018) [18]
*Alistipes*	NSCLC and RCC patients receive Anti-PD-L1 and Anti–PD-1	Improved therapeutic response	Routy et al. (2018 )[18]

*Abbreviations*: *CTLA*-4 cytotoxic T lymphocyte–associated antigen 4, *MM* metastatic melanomas, *NSCLC* non-small-cell lung cancer, *UC* urothelial carcinoma, *PD*-1 programmed cell death protein 1, *PD-L*1 programmed death-ligand 1, *RCC* renal cell carcinoma

Some of the previously published studies identified the same “favourable microbiota” indicative of ICI responsiveness. However, the “favourable microbiota” changes depending on cancer type, suggesting that different tumours may have different “favourable microbiota”. The distinct effect provided by the same microorganisms on the same ICIs is likely have been identified due to sequencing techniques, which can demonstrate the different functions of bacteria based on detailed classification approaches. Further research on other cancer types should be conducted to further reveal the relationship between the gut microbiome and ICI efficacy.

### Chemotherapy

Cancer chemotherapy, defined as treatment with traditional cytotoxic chemotherapeutic agents, has been proven to be influenced by the gut microbiome in murine models, especially therapies with cyclophosphamide (CTX) and oxaliplatin [23, 24, 45]. The effects of cyclophosphamide are partially based on mediation of the antitumour immune response [46]. A previous study showed that the composition of the gut microbiota can be modified by cyclophosphamide, which causes some gram-positive bacteria to translocate into the secondary lymphoid organs, prompting the production of “pathogenic” T helper 17 (pTh17) cells and enhancing the response of the host immune system caused by memory T helper 1 (Th1) cells [23]. The results of this study highlighted the important role of the gut microbiome in cancer immunity and the complicated interaction between the microbiome and chemotherapy [23]. A further study revealed that two specific species in the gut microbiome, *Enterococcus hirae* and *B. intestinihominis*, can influence the clinical benefits of CTX for cancer treatment by reducing regulatory T cells and enhancing the immune response of MHC class I-restricted cytotoxic T cells (CTLs) to the tumour, which eventually alters the tumour microenvironment [45]. Another study demonstrated that commensal bacteria modulate the genotoxicity of platinum compounds by increasing reactive oxygen species (ROS) levels [24]. Recent studies have indicated that resistance to cytotoxic chemotherapeutic agents combined with oxaliplatin and capecitabine in colorectal cancer patients can be enhanced by *Fusobacterium nucleatum* resident in the gut [47]. Consequently, “pharmacomicrobiomics” is an emerging discipline in chemotherapy research [48]. In regard to the side effects of chemotherapy, researchers have found that intestinal microbiota can modulate the adverse drug response of irinotecan-based chemotherapy by reactivating the metabolite of SN-38 glucuronide [49]. In this study, the abundance of *Faecalibacterium prausnitzii* and specific species of *Bacteroides* were significantly different between different cohorts stratified by the metabolism of glucuronide. Inhibiting microbial β-glucuronidases may decrease the serious side effects caused by irinotecan, such as severe diarrhoea, in specific cohorts. Another study on the FOLFOX chemotherapy regimen (5-FU, leucovorin calcium and oxaliplatin) in a colorectal cancer model demonstrated that some microbiome compositions can induce the activation of nuclear transcription factor-κB (NF-κB) and increase the production of interleukin-6 (IL-6) and tumour necrosis factor (TNF), which promotes inflammation and causes mucosal damage [50]. However, the probiotic *Lactobacillus rhamnosus* can help reduce the mucositis induced by chemotherapy by modulating the proinflammatory response and suppressing intrinsic apoptosis in intestinal injury [50]. In general, the aforementioned studies show that the gut microbiome can influence the efficacy of chemotherapy by modulating host immunity, suggesting that the gut microbiome may have a different influence on different traditional cytotoxic chemotherapeutic agents because of the distinct impact of different agents on cancer immunity. Cytotoxic chemotherapeutic agents, such as cyclophosphamide, which are closely related to antitumour immune responses, may be more profoundly affected by the composition of the gut microbiome. In addition to acting as the predicting factor of chemotherapy-related toxicity, particular microbes may become a future therapeutic target to reduce side effects, potentially improve patient compliance and consequently improving the efficacy of cancer treatment. However, more studies are necessary to further determine the association between the microbiome and cancer treatment and to investigate the potential benefits of microbiome modulation.

### Radiation therapy

One of the most indispensable treatments for cancer is radiation therapy. Researchers have found that analyzing the diversity and abundance of the rectal microbiome during cisplatin and radiation therapy (CRT) can predict the clinical outcome of cervical squamous cell carcinoma (CSCC) and concluded that a specific intestinal microbiota may have a positive effect on the efficacy of the treatment, while a specific vaginal microbiota seems to negatively influence the outcome of CRT [51]. Radiotherapy can also induce apoptosis of intestinal cells and cause damage to the gut microbiome composition [52], leading to intestinal inflammation, which may cause diarrhoea and fatigue [53, 54]. The mechanism may rely on the activation of interleukin-1B (IL-1B), which means that the blockade of IL-1B or rebuilding of the gut microbiome system can reduce the damage caused by radiation therapy [55]. A recent study showed that the side effects, such as fatigue, nausea, vomiting and diarrhoea, caused by radiation therapy can be mediated by probiotics such as *Lachnospiraceae* and *Enterococcaceae*, which means that it is likely to reduce the radiation damage caused by the therapy by modulating the gut microbiome [56]. Interestingly, the results of a randomized clinical trial revealed that the combination of probiotics with radiation therapy for patients with nasopharyngeal carcinoma receiving concurrent radiochemotherapy can significantly strengthen host immunity and alleviate the oral mucositis (OM) associated with radiochemotherapy by modifying the gut microbiota [57]. However, there is no clear evidence that the intestinal flora can directly affect the efficacy of radiation therapy.

Although there is not enough evidence showing that the microbiome can directly impact the efficacy of radiation therapy, the association between the side effects of radiation therapy and the gut microbiome makes it possible to modulate the composition of the gut microbiome to reduce radiation therapy-related toxicity, which is likely to improve the prognosis of patients who receive radiation therapy. The mechanism of the association between the host microbiome and the response to and side effects of radiation therapy may be revealed in the future.

### Other therapy

In the case of surgery, researchers have found that the gut microbiome of patients with colorectal cancer is associated with postoperative infections, anastomotic leakage and ileus [58]. Researchers have also found that the gut microbiome of long-term survivors and short-term survivors of pancreatic adenocarcinoma (PDAC) who received surgery show different compositions and abundances, which can be a predictive factor for PDAC patient survival. In addition, the transplantation of specific gut microbiomes from long-term survivors can enhance the immune response to tumours in preclinical murine models [59]. Determining whether modifying the gut microbiome before surgery can improve the prognosis of patients treated by surgery may be a future research direction.

In regard to haematopoietic stem cell transplant (HSCT), previous studies have suggested that in cases of graft versus-host disease (GVHD) induced by HSCT, the gut microbiota is an indispensable factor that can influence the development of GVHD [15, 16, 60]. Peled [60] found that a decreased risk of relapse is associated with *Eubacterium limosum*, while Jenq [15] found that *Blautia i*s related to a decrease in GVHD-related mortality.

Preclinical models have shown that depleting the gut microbiota could maintain the survival of transferred T cells in a cervical cancer model treated with adoptive T cell therapy (ACT), which is dependent on systemic CD8α + dendritic cells (DCs) and interleukin-12 (IL12, [61]). Moreover, in a murine model that received CpG oligonucleotide immunotherapy and interleukin-10 treatment, *A. shahii* was found to improve the response to the treatment [24].

Hormone therapy is also related to the gut microbiome. Sfanos [62] found that people receiving oral androgen receptor axis-targeted therapies (ATT) for prostate cancer had altered gastrointestinal microbiome composition, which may influence the clinical benefits of ATT and potentially influence the efficacy of other therapies, such as immunotherapy, by altering the gastrointestinal microbiota.

The potential clinical effect provided by the gut microbiome on therapy, such as surgery, hormone therapy, and stem cell transplant therapy, and the mechanism of the effect should be further studied in the future.

### The mechanism of gut microbiome-mediated influence on cancer therapy

**Fig. 1:**
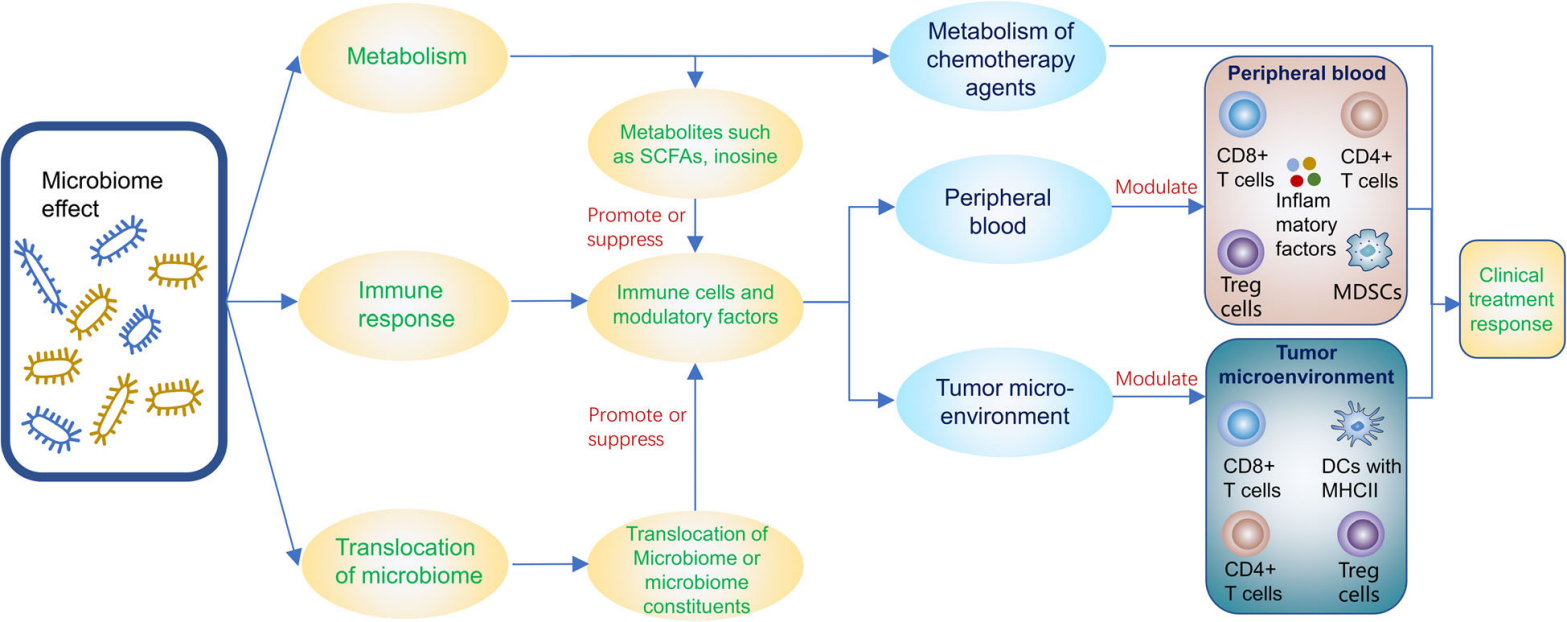
Mechanism of the effects caused by gut microbiome on cancer treatment response. Tregs = regulatory T cells, MDSCs = myeloid-derived suppressor cells, DCs = dendritic cells, SCFAs = short chain fatty acids, Metabolic variations caused by the microbiota can modulate and side effects of cancer therapy, which may depend on the immune system of the host or the metabolism of chemotherapeutic agents in tumour cells, The immune response and inflammation are important aspects of the impact of the micriobiome on cancer treatment efficacy which may be modulate by metabolism or translocation of microbiome and microbiome constituents

Mechanism which has been revealed of the association between gut microbiomes and the efficacy of cancer treatment are summarized in Fig. 1.

### Metabolism

Metabolism of the host or the tumour cell is likely an indispensable factor that may alter the efficacy and side effects of chemotherapeutic agents. The gut microbiota has the potential to influence the metabolism of chemotherapeutic drugs directly or to modulate other small molecules to alter host metabolism and indirectly affect the efficacy of cancer treatment. In regard to the role of small molecules, recent research has revealed that small molecules produced by the gut microbiome are involved in cancer development. Small molecules can modulate antitumour immunity in the liver, and these molecules include lipopolysaccharide (LPS), bile acids (BAs), and lipoteichoic acid (LTA) [63–65]. Researchers have found that anaerobic species are involved in the production of butyrate, a short-chain fatty acid (SCFA) that can enhance the production of IL-10, restrain the activation of NF-κB, and eventually suppress the progression of colitis caused by cancer treatment [48, 66–68]. Moreover, research has proved that the microbiome plays a vital role in the metabolism of chemotherapeutic agents in *Caenorhabditis elegans *(*C. elegans*) [69, 70]. Scott [70] found that the metabolism of bacterial RNA along with vitamins B6 and B9 can modulate the activation of prodrugs. A previous study also showed that inhibiting microbial β-glucuronidases could decrease the side effects induced by irinotecan, including severe diarrhoea caused by dysbiosis, which was related to chemotherapy in specific patients [49]. In brief, metabolism appears to be a necessary aspect of the mechanism by which the microbiome influences the efficacy and side effects of chemotherapy [7].

In regard to the mechanism by which the microbiome influences the efficacy and adverse reactions of immune checkpoint inhibitors, researchers have compared metabolic pathway enrichments and found changes in the metabolic functions of patients with metastatic melanoma receiving immune checkpoint inhibitors [20]. Gopalakrishnan found that anabolic functions predominated in responders to immune checkpoint inhibitors, including amino acid biosynthesis, which likely enhanced host immunity [71], whereas catabolic functions predominated in nonresponders. Recent research has shown that intestinal *B. pseudolongum* could amplify the response to immunotherapy by producing the metabolite inosine [42]. Additionally the decreased function of the gut barrier caused by immunotherapy enhanced the translocation of inosine, which has the ability to activate antitumour T cells.

In summary, metabolic variations caused by the microbiota can modulate the efficacy and side effects of cancer therapy, which may depend on the immune system of the host or the metabolism of chemotherapeutic agents in tumour cells.

### Immune response

The influence of metabolic alterations on cancer therapy appears to be mostly dependent on the modulation of cancer immunity. Researchers have found that the FOLFOX chemotherapy regimen in a colorectal cancer model activated NF-κB, subsequently increasing the production of TNF and IL-6 and consequently leading to local inflammation and mucosal damage [50]. Moreover, the mechanism of the damage caused by radiotherapy may rely on the activation of IL-1B, while blocking IL-1B or rebuilding the gut microbiome system can reduce this damage [55]. Recent research has revealed the relationship between small molecules such as LPS, BAs, and LTA produced by the gut microbiome and antitumour immunity in the liver, as previously reported [63–65]. The production of the metabolite inosine as a result of intestinal *B. pseudolongum* likely leads to the activation of antitumour T cells that express adenosine A2A receptor and enhance immunotherapy [42]. According to previous research, the gut microbiome can not only activate APCs and reinforce the mucosal barrier but can also promote IgA secretion and maintain the balance of Tregs and T-helper-17 (Th17) cells, which eventually promotes the balance of the cytokines that cause or inhibit inflammation [22, 24, 30, 41, 72–76].

In general, metabolism mostly depends on the modulation of cancer immunity to influence the effect of cancer therapy; thus, the immune response and inflammation are important aspects of the impact of the microbiome on cancer treatment efficacy and are worth further study.

### Translocation of the microbiota or microbiome constituents

Previous research has proven that bacteria can pass through the barrier of the intestine and enter the secondary lymphoid organs to modulate the efficacy and toxicity of chemotherapy [23, 45]. Nevertheless, research on the translocation of microbiota showed that this phenomenon can be observed not only in lymphoid organs but also in other organs, including the pancreas. Riquelme performed faecal microbial transplantation (FMT) on a preclinical model of PDAC and examined the abundance of operational taxonomic units (OTUs) in patient donor samples, pre-FMT and post-FMT murine faecal samples, and murine tumours 5 weeks after tumour implantation to validate the association between the tumour microbiome and the gut microbiome [59]. He proved that the gut microbiome could regulate the composition of the tumour microbiome; thus, modification of the gut microbiome can lead to alterations in the tumour microbiome of PDAC patients by direct translocation of the gut microbiome into tumours. However, the dominant mechanism of the tumour microbiome alteration was its modulation by the different microbial landscapes and tumour microenvironments induced by the activation and infiltration of CD8+ T cells into the tumours, which was related to the modulation of the gut microbiome [59]. The gut-lung microbiota axis is another clear avenue of cross-talk that is vital for modulating the host immune response [77]. The relationship between the gut microbiota and the lung microbiota may depend on metabolites produced by the gut microbiota, such as SCFAs, peptidoglycans or LPS, which are associated with the inflammatory response of the lung [78–80]. Researchers have also found that the interaction between the lung and gut microbiomes partially depends on direct microbiome translocation. The migration of immune cells from one site to the other or the release of bacteria-derived immunomodulatory molecules into the circulation system or the lymphatic system also play an important role in this interaction [81–83]. A report on the translocation of gut bacteria-derived products into the circulation revealed that this process could promote liver inflammation associated with liver disease progression and cancer risk [84, 85]. However, the association between the prognosis of liver cancer patients and microbiome translocation has not yet been confirmed. The translocation of the microbiota and the relationship between the gut microbiome and locally resident microbiota or intratumour microbiota in other parts of the body are unclear and should be researched further in the future.

## Cancer therapy and locally resident microbiota or intratumour microbiota

Locally resident microbiota and intratumour microbiota are a current research focus. Locally resident microbiota, especially the microbiota of the gastrointestinal tract and other parts of the digestive system, have been found to be closely related to the carcinogenesis of the resident organ [11, 27–29]. Previous studies have shown that susceptibility to oral squamous cell carcinoma (OSCC) [86], oesophageal cancer [87, 88], gastric cancer [89, 90], gastric diffuse large B cell lymphoma (DLBCL) [91], CRC (colorectal cancer) [92–94], gastric mucosa-associated lymphoid tissue lymphoma (MALT) [95], hepatocellular carcinoma (HCC) [96], pancreatic cancer [97], gallbladder cancer [98], lung cancer [99], breast cancer [100] and prostate cancer [101] is associated with locally resident microbiota. However, the relationship between locally resident microbiota and the efficacy therapy is still under investigation. For colorectal cancer (CRC) patients, the gut microbiome is also the locally resident microbiota at the tumour site. The subsequent discussion is divided into two sections, one concerning patients with CRC and another regarding patients with other cancers.

### Gut microbiome and CRC

As mentioned previously, the gut microbiome can influence the therapeutic effects of immunotherapy, chemotherapy, radiation therapy and surgery for colorectal cancer along with influencing side effects. The influence of the intratumour microbiome of gastrointestinal cancer also plays an indispensable role in the outcome of cancer treatment. In a colon cancer model, Geller found that gemcitabine resistance was induced by intratumour bacteria in a murine model of colon cancer, which could be inhibited by combination treatment with antibiotics [102]. The mechanism may depend on the metabolism of gemcitabine caused by the intratumour microbiome. Intratumoral *Gammaproteobacteria* can influence gemcitabine metabolism and cause gemcitabine resistance. The value of the intratumour microbiome and the metabolism of tumour cells may be another target of future research on other cancers.

### Locally resident microbiomes and other cancers

In PDAC, Riquelme found that the distinct species of the tumour microbiome from the long-term survivors of pancreatic cancer after tumour resection was a prognostic factor for survival, and the transplantation of long-term survivor gut microbiomes could restrict tumour growth by altering the tumour microbiome in murine models [59]. The author demonstrated that gut microbiota could modulate tumour microbiota and influence tumour growth. Researchers have also found that the removal of bacteria resident in pancreatic cancer was related to the immune response to PDAC, and altering the tumour microenvironment by inhibiting the infiltration of myeloid-derived suppressor cells (MDSCs) could enhance the differentiation of M1 macrophages, which stimulated the differentiation of Th1 cells and increased the number of activated CD4+ T cells and CD8+ T cells [7]. Interestingly, the removal of bacteria could even modulate the response of PDAC patients to ICIs by upregulating the expression of PD-1. In regard to the specific mechanism of immune reprogramming, the microbiome of PDAC can differentially activate select Toll-like receptors of monocytic cells to generate a tolerogenic immune program inducing innate and adaptive immune suppression [7].

In prostate cancer patients, Banerjee [103] found distinct microbiome signatures of prostate cancer that were associated with the stages, grades and scores of prostate cancer by analyzing tumour tissue samples, revealing the value of analyzing locally resident flora for predicting the prognosis of prostate cancer patients.

In breast cancer, researchers have found enriched microbes by analyzing the microbiome of breast skin swabs and breast tissue from patients with breast cancer and healthy controls. The enriched microbes in the patients included *Fusobacterium*, *Comamonadaceae*, *Atopobium*, *Gluconacetobacter, Bacteroidetes, Enterobacteriaceae*, *Hydrogenophaga*, *Staphylococcus* and *Bacillus* [100, 104]. However, the association between the microbiome and cancer treatment is still under investigation.

In regard to the microbiome of the airway, a recent study compared brushing samples of bronchi from 24 lung cancer patients and 18 healthy controls. The samples collected from the patients included the unilateral lobar tumour and paired samples from the cancerous site and the opposite site of the tumour, i.e., the noncancerous site [105]. The author demonstrated that microbiota profile of the samples collected from the cancerous site of lung cancer patients was different from that of the samples collected from healthy controls, including the samples from the noncancerous site and from the healthy controls, which showed a lower microbial diversity than that of the noncancerous site and healthy controls. Tumour tissue had a higher abundance of *Streptococcus* and *Neisseria* than normal tissue, while *Staphylococcus* and *Dialister* were found to reside in normal tissue more frequently. There was a trend that the abundance of microbiota changed gradually from normal tissue to noncancerous site tissue to cancerous tissue in lung cancer patients, suggesting that the microbiota of the lung can clearly affect the tumour microenvironment, which is not restricted to the cancerous site but involves the whole lung and is likely related to cancer progression and patient prognosis [105]. The nasopharynx is the upper part of the pharynx, an indispensable part of the airway. The association between the nasopharynx microbiome and cancer treatment has not been well studied and meaningful results may be obtained in the future.

On the one hand, the intratumour microbiome is capable of decreasing the effective concentration of chemotherapeutic agents and the expression of major histocompatibility complex (MHC) class I as well as increasing the number of MDSCs. Also, the tumour microbiome can induce alternative immune checkpoints and restrict the clonal expansion of lymphocytes [72, 73]. On the other hand, the tumour microbiome can not only directly engage the innate immune system but can also produce more anti-inflammatory cytokines and increase the expression of targetable checkpoint molecules, which is likely to be beneficial to cancer immunity [72, 73]. The influence of the tumour microbiome on the immune microenvironment is a double-edged sword and worthy of further study in a variety of cancers. In summary, the effect of locally resident microbiota or intratumour microbiota on cancer therapy has not been well studied. Although the function of some locally resident or intratumour bacteria has been confirmed in some individual cases, these studies mainly focused on the gut. In cancers of other areas of the digestive tract, there is not enough evidence for the interaction between the efficacy of cancer therapy and the locally resident microbiome. However, there is some evidence that locally resident microbiota are associated with local inflammation and the progression of cancer, indicating that patient prognosis could be improved through microbiome modification. In general, the bacteria resident in multiple areas of the human body seem to participate in the various stages of tumour development, the mechanism of which may rely on innate and specific immune responses and remains unknown.

## Discussion and prospective

### Our findings

We discussed the potential role of gut microbiota and locally resident microbiota in cancer therapy and three major probable mechanisms underlying this relationship. We found that the influence of metabolism mostly depended on modulating cancer immunity to affect cancer therapy [48, 66–68], while some microbiota can directly modulate chemotherapeutic agent metabolism [102]. Microbiota translocation also depends on host metabolism and the immune system influence the efficacy of cancer immunity [78–83]. The efficacy of several cancer therapies, including radiation therapy, surgery, chemotherapy, and other kinds of therapy, such as cell transplant therapy, has been found to be associated with the gut microbiome or the locally resident microbiota or intratumour microbiota. The association between microbiota and cancer therapies tends to be influenced by the association between the therapies and the host immune response. In other words, if the response of the therapy is closely related to the immune response, the therapy tends to be greatly affected by the microbiome, especially the gut microbiome, which has been proven to be closely related to the host immunity of pathogens and cancer [17, 30, 106]. Cancer immunotherapy is dependent on host immunity and may be more likely to be affected by the microbiota. Immune checkpoint blockade beneficial for improving survival rates in many cancers and represents a breakthrough in cancer therapy [32]. The relationship between the efficacy of immune checkpoint blockade and the human microbiome has been confirmed for many malignancies [13, 14, 18–22, 41, 42]. Different tumours may have different “favourable microbiota”, while the same kind of microbiota may have distinct effects on the same ICIs for different tumours. Instead of continuing research on the efficacy of specific microbiomes, research on the mechanisms and downstream pathways associated with immune response differences caused by microbiome differences may yield benefits.

### Methods to modulate the host microbiome

The microbiome could be a beneficial target to improve cancer response to treatment. The major methods for modulating the microbiome to date include FMT [107–109], probiotics and prebiotics [3, 57, 108, 110], and diet control [111–113]. FMT-a therapy that has been used for inflammatory bowel disease [114], has been confirmed to improve the efficacy of cancer treatment [20, 59] in murine models and to extenuate ICI-associated colitis in clinical practice [107]. As shown by V. Gopalakrishnan [20], mice transplanted with stools from human responders with metastatic melanoma treated with ICIs showed improved responses to anti-PD-L1 therapy in contrast to the response of mice that were transplanted with stools from nonresponders. The median fold change in the tumour volume in mice transplanted with responder stools was 0.18, while the median fold change of tumour volume in mice transplanted with nonresponders stools 1.52 (P<0.01), showing the promising efficacy of FMT. Routy [18] found that transplantation of *Akkermansia Muciniphila*(*A. muciniphila)* or *A. muciniphila* plus *Enterococcus hirae(E. hirae)* reversed the poor efficacy of PD-1 blockade in germ-free mice treated by FMT with stools from human nonresponders. The tumour size of the *A. muciniphila* or *A. muciniphila* plus *E. hirae*-treated mice was much smaller than the tumour size of mice in the control group (*P* = 0.038 in the *A. muciniphila* group and P<0.001 in the *A. muciniphila* plus *E. hirae* group). Moreover, patients with ICI-associated colitis have been successfully treated with FMT [107]. Two patients with poor response to corticosteroids and anti-TNF-α agents were treated successfully by FMT, as confirmed by endoscopic evaluation and immunohistochemistry. FMT is becoming the most feasible way to modulate the gut microbiome. Transplantation of the entire ecosystem directly ensures the efficacy of the modulation. However, the complexity of the techniques, donor selection and persistence time should be considered. More clinical trials of faecal microbiota transplantation should be performed to address these problems. Probiotics have been used for the prevention of many diseases [115–118]. Although the simplicity of adjuvant therapy allows for long-term use, the objective efficacy of the therapy is difficult to assess and monitor; thus, more research is required to address these issues. The results of a recent randomized clinical trial showed that the combination of probiotics can significantly decrease the occurrence rate of high-grade OM caused by chemoradiotherapy compared to that observed in patients administered a placebo. The incidences of grade 0, 1, 2, 3 OM in the placebo group and the probiotic combination group were 0, 0, 54.29, 45.71 and 12.07%, 55.17, 17.24, 15.52% (P<0.0001), showing the feasibility of modulating the efficacy or side effects of cancer treatment by probiotics [57]. In regard to therapy dependent on the diet, although some reports show that specific diets are associated with carcinogenesis or the efficacy of cancer treatment [113, 119–121], no prospective trial has identified the specific relationship between treatment and diet. Moreover, patients may not adjust well to the new diet, reducing compliance. Diet modulation is likely to be used as a supplement to therapy in the future.

### Direction of future research

The influence of the microbiome on the efficacy of treatment and the relationship between the host microbiome and cancer treatments efficacies should be explored in the future, especially in the context of immune checkpoint blockade. Mechanistic studies will likely identify targets for cancer therapy and allow for the modulation of the efficacy of cancer therapy directly without modulating the microbiome.

Research on the interaction between the immune response and microbiome should be further conducted on more cancer types to uncover the mechanism of the immune response, which may be modulated without altering the microbiome in the future. Modulating inflammation or the immune response by modulating inflammatory factors may be a better way to enhance cancer treatment efficacy, inspired by the effects of the microbiome.

Specific metabolites, such as inosine, can modulate the efficacy of cancer treatment, and this effect is not dependent on the microbiome. The metabolite inosine produced by the microbiome has been shown to modulate the efficacy of cancer therapy without modulating the microbiome in murine models [42], which may be used as an adjuvant agent for various kinds of cancer therapy in the future. The application of specific metabolites produced by the microbiome may replace the role of the microbiome. The efficacy should be studied further in more kinds of cancer and in clinical trials. Future research on the detailed mechanism of the effects caused by inosine or other metabolites should be performed as well, which may depend on research on the interaction between the immune response and microbiome-derived metabolites.

In addition to mechanistic research on metabolism and the immune response, finding a way to modulate the translocation of microbiota and microbiota-derived metabolites may be another future research direction to investigate the relationship between gut microbiota and locally resident microbiota or intratumour microbiota, which may be another important mechanism of the effects caused by the gut microbiome on cancer treatment.

Mechanistic studies of the influence of the locally resident microbiome on the tumour microenvironment should be performed by studying the interaction between the immune response and metabolism. Performing research on the modulation of locally resident microbiome may be a simple way to enhance the cancer treatment efficacy up to now.

## Conclusion

We found that the microbiome can modulate the efficacy of cancer therapy by modulating metabolism to enhance or suppress the immune response to the tumour or by modulating the metabolism of antitumour agents. Some microbiomes can modulate the immune response directly, and the specific downstream pathway remains unknown. Future studies may concentrate on identifying unknown pathways, developing a feasible plan for the modulation of the gut microbiome and identifying the mechanism of modulation to identify treatment targets. The interaction between the host immune response and the microbiome should be further studied to identify the specific pathway involved in the activation of immune cells caused by the human microbiome. In addition, the mechanism of the effects induced by the human microbiome in the various stages of tumour development, such as carcinogenesis and metastasis, has not yet been well studied and the interaction between the microbiome, metabolism, immune response and translocation of the microbiome should be further studied in future. Future research on the relationship between host immunity and the host microbiome will create a brighter future cancer patients.

## Data Availability

Not applicable.

## Abbreviations

ICI: Immune checkpoint inhibitors
PD-1: Programmed death receptor
PD-L1: Programmed death receptor ligand
CTLA-4: cytotoxic T lymphocyte-associated protein 4
MM: Metastatic melanomas
NSCLC: Non-small-cell lung cancer
UC: Urothelial carcinoma
RCC: Renal cell carcinoma
CTX: Cyclophosphamide
pTh17: “pathogenic” T helper 17
Th1: T helper cells
CTL: Cytotoxic T cells
ROS: Reactive oxygen species
FOLFOX: 5-FU, Leucovorin Calcium and oxaliplatin
NF-κB: Nuclear transcription factor-κB
TNF: Tumour necrosis factor
IL-6: Interleukin-6
CRT: Cisplatin and radiation therapy
CSCC: Cervical squamous cell carcinoma
IL-1B: Interleukin-1B
OM: Oral mucositis
PDAC: Pancreatic adenocarcinoma
HSCT: Haematopoietic stem cell transplant
GVHD: Graft versus-host disease
ACT: Adoptive T cell therapy
DCs: Dendritic cells
IL12: Interleukin-12
ATT: Axis-targeted therapy
OSCC: Oral squamous cell carcinoma
DLBCL: Diffuse large B cell lymphoma
CRC: Colorectal cancer
MALT: Mucosa-associated lymphoid tissue
HCC: Hepatocellular carcinoma
LPS: Lipopolysaccharide
BAs: Bile acids
LTA: Lipoteichoic acid
SCFA: Short-chain fatty acid
MDSC: Myeloid-derived suppressor cells
APC: Antigen presenting cells
MHC: Major histocompatibility complex
FMT: Faecal microbial transplantation
OTU: Operational taxonomic unit
PFS: Progression-free survival
OS: Overall survival
ATB: Antibiotics
*C. elegans*: *Caenorhabditis elegans*
*A. muciniphila*: *Akkermansia Muciniphila*
*E. hirae*: *Enterococcus hirae*
